# Effects of fluid administration on renal perfusion in critically ill patients

**DOI:** 10.1186/s13054-015-0963-0

**Published:** 2015-06-12

**Authors:** Mouhamed Djahoum Moussa, Sabino Scolletta, David Fagnoul, Pierre Pasquier, Alexandre Brasseur, Fabio Silvio Taccone, Jean-Louis Vincent, Daniel De Backer

**Affiliations:** Department of Intensive Care, Erasme University Hospital, Université Libre de Bruxelles, Brussels, Belgium

## Abstract

**Introduction:**

Fluid administration is a first-line therapy for acute kidney injury associated with circulatory failure. Although aimed at increasing renal perfusion in these patients, this intervention may improve systemic hemodynamics without necessarily ameliorating intrarenal flow distribution or urine output. We used Doppler techniques to investigate the effects of fluid administration on intrarenal hemodynamics and the relationship between changes in renal hemodynamics and urine output. We hypothesized that, compared to systemic hemodynamic variables, changes in renal hemodynamics would better predict increase in urine output after fluid therapy.

**Methods:**

We measured systemic hemodynamic variables and performed renal interlobar artery Doppler on both kidneys before and after volume expansion in 49 adult patients with acute circulatory failure. We measured systolic and diastolic velocities and computed the resistivity index (RI). We recorded urine output for 3 h before and after the fluid challenge.

**Results:**

Fluid administration resulted in a small but consistent decrease in RI (from 0.73 ± 0.09 to 0.71 ± 0.09, *p* < 0.01). There was a concomitant increase in mean arterial pressure (from 75 ± 15 to 80 ± 14 mmHg, *p* < 0.01), pulse pressure (49 ± 19 to 55 ± 19 mmHg, *p* < 0.01) and urine output (55 ± 76 to 81 ± 87 ml/hour, *p* < 0.01). Changes in RI were negatively correlated with changes in urine output and mean arterial pressure but not in pulse pressure. The increase in urine output was predicted by changes in RI but not by changes in systemic hemodynamics.

**Conclusions:**

Changes in renal hemodynamics during a fluid challenge can be observed by Doppler ultrasonography before urine output increases. Moreover, these changes are better predictors of an increase in urine output than are mean arterial pressure and pulse pressure.

## Introduction

Imbalance in oxygen delivery and oxygen demand is common in critically ill patients, especially those with acute circulatory failure of septic, hypovolemic or cardiogenic origin. Acute kidney injury (AKI) is a frequent complication of circulatory failure and associated with increased morbidity and mortality [1, 2]. Fluid resuscitation is a first-line therapy used to restore oxygen delivery to the organs and prevent AKI [1–3] and yet the renal effects of fluid resuscitation are not easily assessed. Excessive fluid administration may have harmful effects, and mortality rates are increased in patients with AKI who develop a positive fluid balance [4–6]. Hence, it could be important to identify those patients in whom renal hemodynamics improve in response to fluids. Although fluids are often given to patients with AKI with the aim of increasing renal perfusion, physicians generally evaluate the effectiveness of fluid therapy by looking at systemic hemodynamic variables, such as mean arterial pressure (MAP) or cardiac output, because the increase in urine output may be delayed or inconsistent and the decrease in serum creatinine may be even slower. A direct evaluation of renal hemodynamics would be valuable, but is not easily obtained at the bedside.

Recently, interlobar artery (ILA) resistivity index (RI), a reliable surrogate of intrarenal vascular tone measured by renal interlobar artery Doppler (RIAD), has been proposed for bedside evaluation of intrarenal hemodynamics in critically ill patients [7–9]. This index has been used to identify septic patients who develop AKI several days after ICU admission [7], and to separate patients who develop transient versus persistent AKI [10]. A recent meta-analysis of nine trials suggested that elevated RI was significantly associated with persistent AKI [11]. This variable has also been successfully used in critically ill patients to evaluate changes in renal hemodynamics following various therapeutic interventions, such as norepinephrine infusion, fluid challenge, mild hypoxemia or paracentesis [8–13]. However, none of these studies compared the effectiveness of changes in systemic and in intrarenal hemodynamics to predict changes in urine output after a fluid challenge.

The aim of this study was to evaluate changes in ILA RI, using Doppler techniques, during volume expansion in critically ill patients and, in addition, to compare the performance of these changes with that of changes in systemic hemodynamics for the prediction of subsequent variations in urine output. We hypothesized that dynamic analysis of intrarenal hemodynamics using RIAD following a fluid challenge may help to identify responders to fluids better than systemic hemodynamics.

## Methods

### Setting and patients

Erasme University Hospital Ethics Committee approved this interventional study (P2011/125) and informed consent was obtained from the patients or their next of kin. In a convenience sample of patients admitted between April 2011 and March 2012, three groups were studied: two control groups and an interventional group. The control groups included ICU patients with stable systemic hemodynamics receiving no specific intervention. In the first control group, A, we evaluated intra-observer variability in RIAD measurements by performing three measurements 15 min apart. The ultrasound measurements for this assessment were performed by the same technician but the calculations of RI were performed by a statistician not involved in the measurements or the design of the study. In control group B, we assessed stability of the Doppler variables over time, by performing two measurements 60 min apart.

The interventional group consisted of patients with acute circulatory failure who required a fluid challenge. Acute circulatory failure was defined as the association of arterial hypotension (systolic arterial pressure <90 mmHg or MAP <65 mmHg) or need for vasopressor to correct hypotension, and/or oliguria defined as urine output <0.5 ml/kg/hour with at least one of the following criteria: impaired mentation, mottled extremities, arterial lactate >2 mmol/l, and superior vena cava (ScvO_2_) or mixed (SvO_2_) venous oxygen saturations <70 % or <65 %, respectively. Exclusion criteria were age <18 years, atrial fibrillation or frequent ventricular arrhythmias, history of renal transplantation or stenosis of renal arteries, end-stage renal disease, known unilateral kidney, pregnancy, and body mass index >40 kg/m^2^. Patients were also excluded from the study if they were given diuretics or there were any changes in vasopressor administration during the study period. AKI was defined as an increase in serum creatinine ≥0.3 mg/dl over 24 h and/or a 1.5-fold increase in baseline serum creatinine over 7 days, and/or oliguria with a reduction in urine output <0.5 ml/kg/hour over 6 h [14].

### Systemic hemodynamics and renal Doppler measurements

Monitoring included continuous electrocardiogram and heart rate recording and invasive measurement of MAP and pulse pressure (PP) through a radial or femoral catheter. We also measured arterial lactate and ScvO_2_/SvO_2_. One investigator (MDM) performed all Doppler measurements using a convex 2–5.5 MHz probe (4C RS, General Electric Healthcare, Diegem, Belgium), with the transducer applied in a lateral or postero-lateral view to visualize the kidneys. Intrarenal vessels were observed using color Doppler, then a pulsed Doppler signal was obtained on the ILA. Three consecutive measurements displaying at least three ILA Doppler waves each (corresponding to three cardiac beats) were performed in the upper, middle and lower poles of both kidneys and averaged. Failure to meet these criteria or a difference of more than 5 % in RI between the kidneys was considered as a failure in measurement. Systolic (SV) and diastolic (DV) velocities were measured on each wave. Interlobar RI was calculated using the formula: RI = (SV – DV)/SV.

### Study protocol

**Fig. 1:**
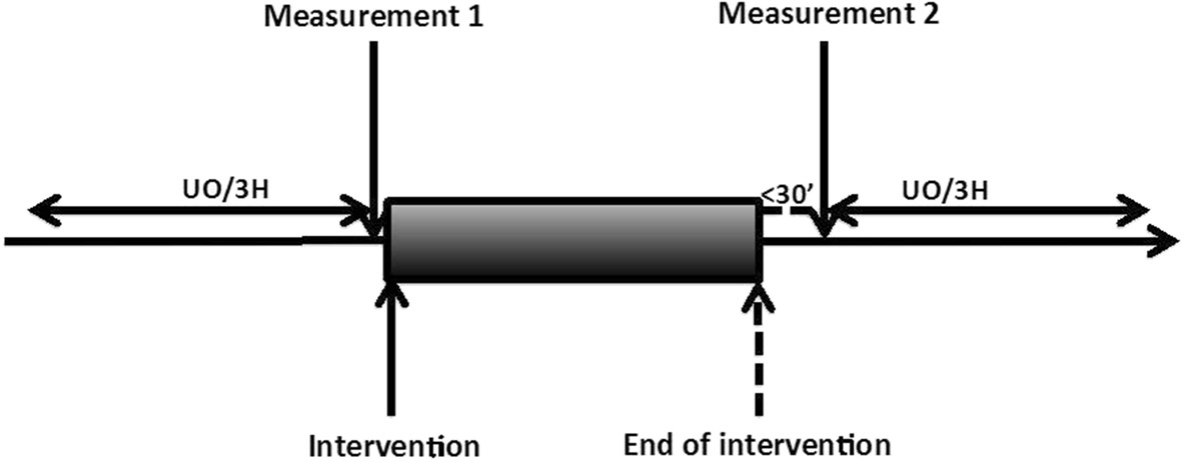
Summary of the study design. The intervention was a fluid challenge that consisted of infusion of a minimum volume of 500 ml at a minimum rate of 1,000 ml/hour. Interlobar artery Doppler variables, blood pressure, heart rate and urine output were recorded before and after the fluid challenge and stabilization of hemodynamic variables. *UO/3H*: urine output volumes measured over 3 hours

Fluid challenge consisted of a minimum volume of 500 ml crystalloid (NaCl 0.9 %, Hartman’s or Plasmalyte solution (Baxter, Lessines, Belgium) or hydroxyethyl starch 6 % (Voluven, Fresenius, Bad Homburg, Germany)) infused at a minimum rate of 1,000 ml/hour under arterial pressure guidance [15]. Fluid administration was stopped when MAP reached a predetermined goal (generally MAP >65 mmHg, and/or MAP and/or stroke volume increase >10–15 % compared to baseline) and/or central venous pressure increased >15 mmHg. Systemic hemodynamic variables were recorded and renal Doppler measurements were performed before and at the end of the fluid challenge. The urine output volumes over the 3 h preceding and following the fluid challenge were recorded (Fig. 1).

### Statistical analysis

Our primary objective was to describe whether the fluid challenge induced changes in RI. Our secondary objective was evaluation of the relationship between changes in renal Doppler and urine output. We evaluated the performance of renal RI, compared to MAP and PP, to predict an increase in urine output after fluid administration (“renal responsiveness”) using three predefined arbitrary thresholds: increase in urine output by at least 0.1 ml/kg/hour, 0.2 ml/kg/hour and 0.3 ml/kg/hour. The 0.3 ml/kg/hour threshold was used because such an increase in urine output implies improvement to a better class of the AKIN [14] or RIFLE [16] classifications.

We also separated patients into systemic hemodynamic responders (increase in MAP or PP >10 % after fluid challenge) and non-responders to fluid challenge. Change in RI was calculated as the difference in RI divided by baseline RI. Similarly, changes in MAP and PP were calculated as the absolute difference observed after fluid challenge divided by the baseline value.

Data distribution was verified using a Kolmogorov-Smirnov test. Continuous variables are presented as median (interquartile range) or mean ± standard deviation. Baseline characteristics of responders and non-responders were compared using Fisher’s exact test or chi square test and Mann Whitney’s rank sum test as appropriate. In control group A, an intraclass correlation was used to evaluate variability of measurements. In control group B, we calculated the coefficient of variation (CV) of RI and computed the coefficient of error (CE) using the formula CE = CV/√n, where n is the number of measurements in each patient. The precision of the RI measurements was calculated as two CE for average measurements with a level ≤10 % considered acceptable as recommended [17]. We computed the least significant change (LSC), which is the lowest degree of change between two measurements over time that must be exceeded to consider a change to be significant (confidence interval 95 %), using the equation LSC = CE × 1.96 × √2.

In the intervention group, paired Student’s *t* test or Wilcoxon rank test were used for paired comparisons. Changes in RI and in MAP, PP and urine output were examined for correlations using Pearson’s linear correlation coefficient. In subgroups of patients (AKI or no-AKI, oliguria or no-oliguria, fluid challenge systemic hemodynamic responder and non-responder), median values were compared using the Wilcoxon rank test and linear correlation was verified using Spearman’s correlation coefficient. A mix model analysis was performed considering subgroups and the time of measurement.

Receiver operating characteristic curves were generated to test the predictive discrimination of renal responders and non-responders to fluid therapy. Areas under the curve of changes in Doppler variables and of changes in systemic hemodynamic variables were generated and tested for significant differences using the DeLong test. These analyses were also performed for systemic hemodynamic fluid responsiveness. Significance was assumed for a two-sided *p* value < 0.05. All statistical analyses were performed using SPSS 19 for Windows (IBM SPSS Statistics, Chicago, IL, USA).

## Results

**Fig. 2:**
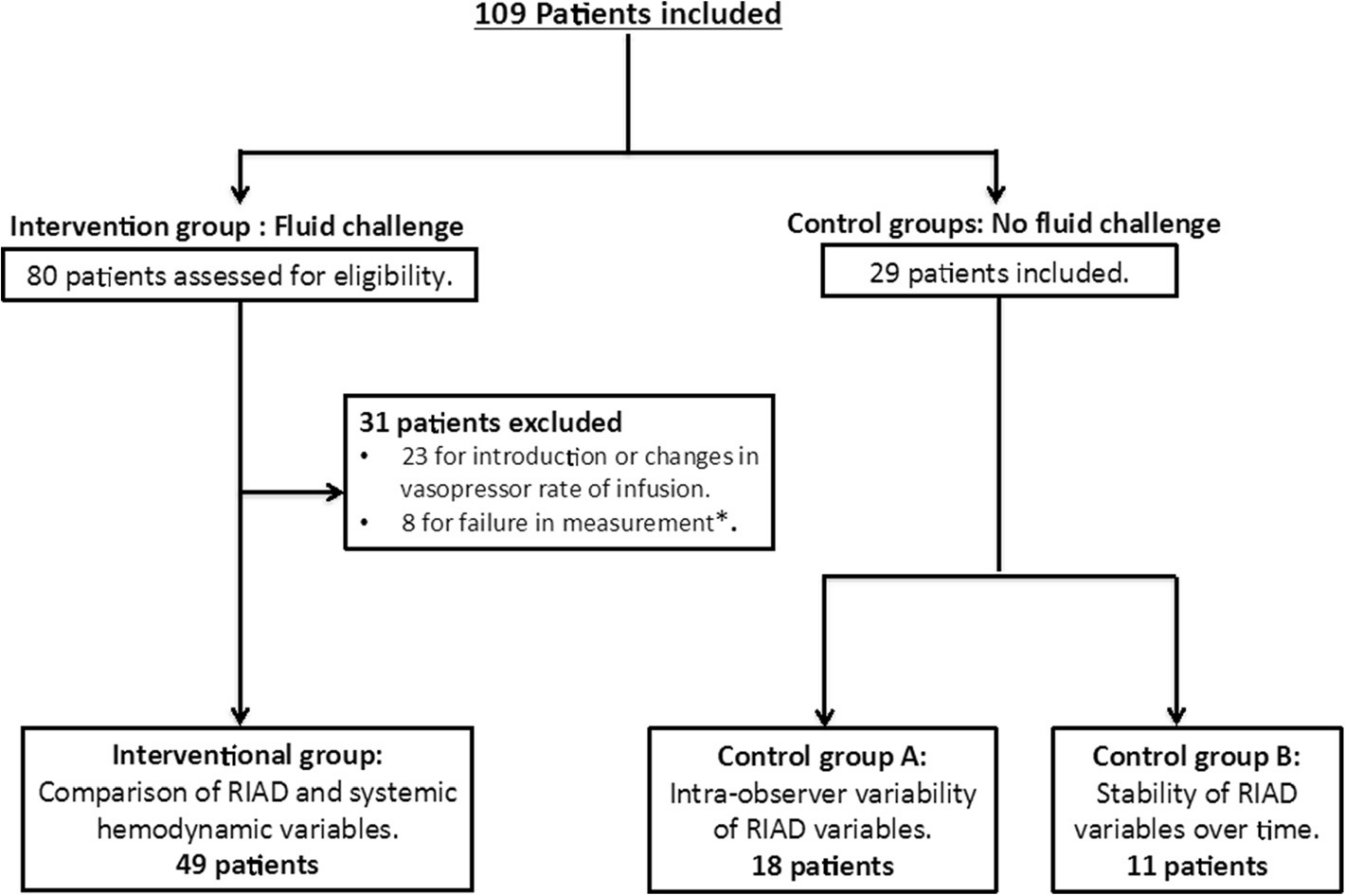
Study Consort diagram. *Six patients had two Doppler waves instead of three or more, two patients had >5 % difference in RI between the kidneys. *RIAD* renal interlobar artery Doppler

**Fig. 3:**
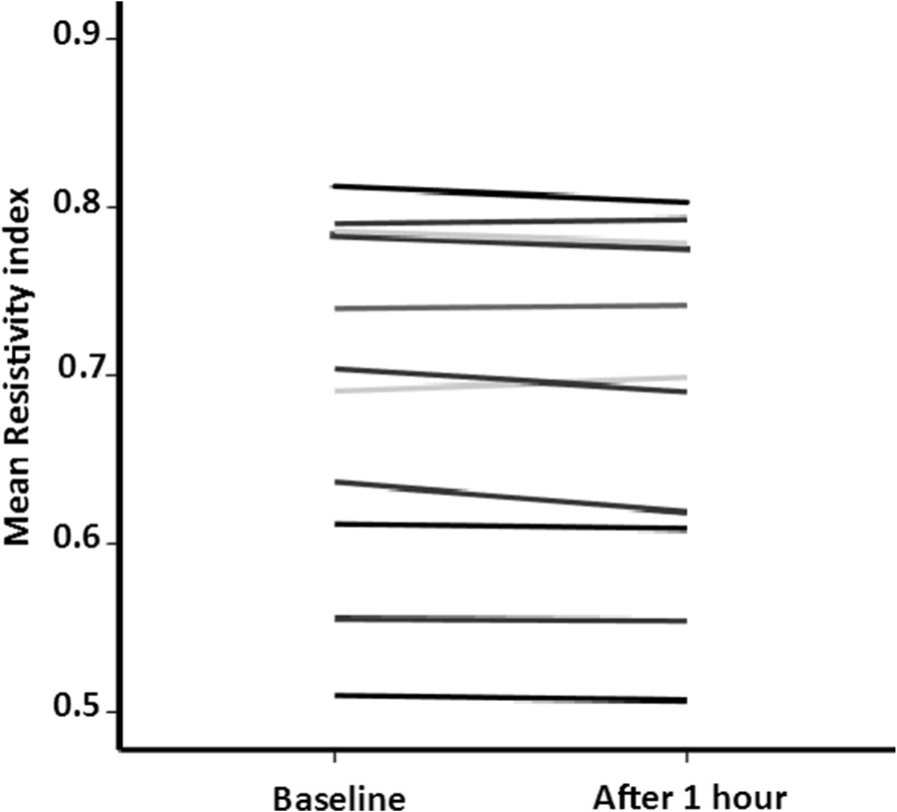
Individual changes in RI over time in the non-intervention group (Control group B). Each line corresponds to the change in RI in one stable ICU patient. Measurements were performed at baseline and 1 hour later in stable hemodynamic conditions with no intervention

We studied 29 patients in the control groups (A, n = 18 and B, n = 11) to evaluate the variability of the renal Doppler measurements (Fig. 2): RI remained stable over 1 h of observation (Fig. 3), with a precision level of 0.9 % (0.3–1.2 %), far below the usually admitted 10 % level. The LSC was 0.01. The intraclass correlation coefficient of three RI measurements 15 min apart was excellent (0.998, *p* < 0.001).

**Fig. 4:**
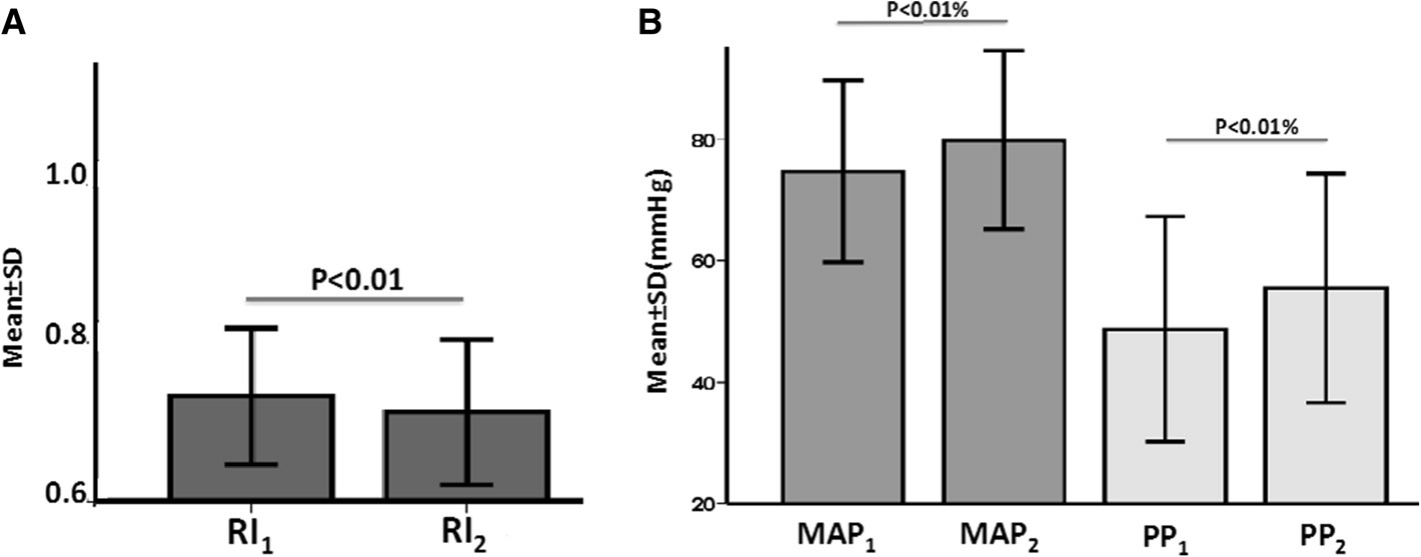
Changes in hemodynamic variables before and after fluid challenge. **a** Changes in resistivity index (*RI*). **b** Changes in mean arterial pressure (*MAP*) and in pulse pressure (*PP*). The *y*-axes show the mean values ± standard deviation. MAP_1_, PP_1_, RI_1_ are values before fluid challenge and MAP_2_, PP_2_, RI_2_ are values after fluid challenge

Of the 80 patients assessed for inclusion in the intervention group, 31 were excluded (eight for failure to obtain adequate renal Doppler measurements (six patients had two Doppler waves instead of at least three, and two patients had >5 % difference in RI between the kidneys) and 23 for concurrent changes in vasoactive support) (Fig. 2). The demographic characteristics of the remaining 49 patients are presented in Table 1. At baseline, 20 (41 %) patients had arterial hypotension, 7 (14 %) had normotension under vasopressor therapy, and 27 (55 %) had oliguria (of these 22 had oliguria without hypotension or vasopressor therapy). The arterial lactate level was elevated in 20 (53 %) patients and ScvO_2_ or SvO_2_ was low in 13 (68 %). The patients received 1,224 ± 717 ml of fluids, primarily as crystalloids (Table 2). Fluid administration resulted in increases in MAP from 75 ± 15 to 80 ± 14 mmHg (*p* < 0.01; Fig. 4), and PP from 49 ± 19 to 55 ± 19 mmHg (*p* < 0.01; Fig. 4). RI decreased concurrently from 0.73 ± 0.09 to 0.71 ± 0.09 (*p* < 0.01; Fig. 4) and urine output increased from 55 ± 76 to 81 ± 87 ml/hour (*p* < 0.01).

**Table 1:** Demographic characteristics of patients at baseline (interventional group, n = 49)

Characteristic	Value
Age (years)	62 ± 16.4
Male, n (%)	34 (69)
Weight (Kg)	80 ± 16
Mechanical ventilation, n (%)	33 (67.3)
Vte (ml)	499 ± 82
PEEP (cmH_2_O)	6 ± 1.2
PaO_2_ (mmHg)	91 ± 25
FiO_2_ (%)	50 ± 20
Type of patient, n (%)	
Medical	26 (53)
Surgical	23 (47)
Type of acute circulatory failure, n (%)	
Septic	24 (49)
Hypovolemic	27 (55)
Cardiogenic	4 (8)
Oliguria	27 (55)
Acute Physiology and Chronic Health Evaluation score	22.4 ± 7.8
Sequential Organ Failure Assessment score	8.8 ± 5.3
RIFLE score	
Risk, n (%)	13 (26.5)
Injury, n (%)	11 (22.4)
Failure, n (%)	7 (14.3)
Loss, n (%)	6 (12.2)
ESKD, n (%)	1 (2)

Results given as mean ± standard deviation, or frequency (percentage). *FiO*_*2*_ inspiratory oxygen fraction, *PaO*_*2*_ arterial oxygen pressure, *PEEP* positive end-expiratory pressure, *RIFLE* Risk (R), Injury (I), Failure (F), Loss (L), End-stage kidney disease (*ESKD*), *Vte* expiratory tidal volume

**Table 2:** Hemodynamic characteristics of renal responders and non-responders at baseline

Variables	All	0.1 ml/kg/hour		0.2 ml/kg/hour		0.3 ml/kg/hour	
		R	NR	R	NR	R	NR
N	49	32	16	27	21	25	23
Heart rate (bpm)	98 ± 23	98 ± 22	97 ± 27	101 ± 22	94 ± 25	101 ± 22	94 ± 24
Systolic arterial pressure (mmHg)	109 ± 21	113 ± 24	102 ± 13	116 ± 25*	101 ± 12	117 ± 25*	101 ± 12
Diastolic arterial pressure (mmHg)	58 ± 13	60 ± 15	53 ± 8	63 ± 15*	52 ± 8	64 ± 15*	52 ± 8
Mean arterial pressure (mmHg)	75 ± 16	79 ± 17	70 ± 9	80 ± 17*	68 ± 8.9	82 ± 17*	68 ± 8.9
Pulse pressure (mmHg)	48 ± 19	50 ± 20	46 ± 17	51 ± 22	47 ± 15	50 ± 22	47 ± 15
Resistivity index	0.73 ± 0.09	0.71 ± 0.09*	0.77 ± 0.06	0.70 ± 0.09*	0.77 ± 0.63	0.70 ± 0.09*	0.76 ± 0.06
Systolic velocity (cm/s)	42.8 ± 17.7	42.2 ± 16.4	45.6 ± 19.9	41.7 ± 14.3	45.3 ± 21.2	43.3 ± 13.5	43.3 ± 21.4
Diastolic velocity (cm/s)	11.4 ± 5.9	12.2 ± 6.4	10.2 ± 4.7	12.7 ± 6.5	10 ± 4.6	13.2 ± 6.6	9.7 ± 4.5
Mean velocity (cm/s)	21.8 ± 9.1	27.2 ± 10.7	27.9 ± 11.8	27.2 ± 9.9	27.7 ± 12.6	28.2 ± 9.5	26.5 ± 12.6
Urine output (ml/kg/hour)	0.69 ± 1.04	0.73 ± 0.85	0.65 ± 1.39	0.79 ± 0.88	0.61 ± 1.24	0.83 ± 0.9	0.57 ± 1.19
Lactate, mean value (mmol/l)	2.8 ± 2.6	2.7 ± 2.5	3.1 ± 3.1	2.8 ± 2.6	2.8 ± 2.8	2.9 ± 2.7	2.8 ± 2.6
Number, n (%)	41 (83.7)	28 (87.5)	12 (75)	23 (85.2)	17 (80.1)	21 (84)	19 (82.6)
ScvO_2_, mean value (%)	65.6 ± 9.2	66 ± 7.5	64.7 ± 12.4	65.6 ± 7.8	65.5 ± 11.5	65.3 ± 8.3	65.8 ± 10.7
Number, n (%)	16 (32.7)	10 (31.3)	6 (37.5)	9 (33.3)	7 (33.3)	8 (32)	8 (34.8)
Norepinephrine rate (μg/kg/minute)	0.31 ± 0.35	0.19 ± 0.14	0.23 ± 0.4	0.22 ± 0.21	0.33 ± 0.39	0.22 ± 0.20	0.33 ± 0.39
Number, n (%)	17 (35)	7 (15)	9 (19)	3 (6)	13 (27)	3 (6)	13 (27)
Fluids crystalloids, n (%)	41 (85)	29 (91)	13 (81)	25 (93)	17 (81)	23 (92)	19 (83)
Colloids, n (%)	14 (29)	8 (25)	3 (19)	6 (22)	5 (24)	5 (20)	6 (26)
Mixed, n (%)	6 (13)	5 (16)	0 (0)	4 (15)	1 (5)	3 (12)	2 (9)
Volume (ml)	1,224 ± 717	1,273 ± 555	1,125 ± 979	1,361 ± 534	1,048 8 ± 83	1,390 ± 545	1,044 ± 842

One patient received norepinephrine, had urinary retention and was not defined as a responder or non-responder. Baseline mixed venous saturation measured in two patients was 49 and 59 %, respectively. Results are shown as mean ± standard deviation or frequency (percentage). *Statistically significant difference versus non-responders for that threshold. *n* number of patients or measurements, *NR* non-responders, *ScvO*_*2*_ central venous oxygen saturation, *R* responders

Changes in Doppler RI correlated significantly with changes in urine output (r = −0.322, *p* = 0.03) and changes in MAP (r = −0.443, *p* < 0.01) but not with changes in PP. We found no correlation between changes in urine output and changes in MAP or PP. The total amount of fluid was correlated to changes in MAP (r = 0.32, *p* = 0.027) and PP (r = 0.33, *p* = 0.02) but not to changes in RI (r = 0.24, *p* = 0.1) or urine output (r = 0.17, *p* = 0.24).

At baseline, RI predicted an increase in urine output following a fluid challenge at all thresholds and MAP predicted an increase at thresholds of 0.2 ml/kg/hour and 0.3 ml/kg/hour, but not 0.1 ml/kg/hour (Table 3). During fluid administration, changes in RI but not changes in systemic hemodynamic variables predicted an increase in urine output at any of the three thresholds (Table 3). The diagnostic performances of changes in RI to predict an increase in urine output are shown in Table 4.

**Table 3:** Performances of RI, MAP, PP and changes in these variables to predict a significant increase in urine output

	Threshold								
	0.1 ml/kg/hour			0.2 ml/kg/hour			0.3 ml/kg/hour		
Variables	AUC	*p*	95 % CI	AUC	*p*	95 % CI	AUC	*p*	95 % CI
RI	0.68	0.04	0.52–0.84	0.73	<0.01	0.58–0.87	0.68	0.03	0.53–0.87
MAP	0.65	0.11	0.49–0.80	0.73	0.01	0.59–0.87	0.76	0.00	0.62–0.89
PP	0.57	0.46	0.40–0.74	0.55	0.52	0.39–0.72	0.55	0.56	0.38–0.72
deltaRI	0.73	0.01	0.59–0.88	0.74	<0.01	0.60–0.88	0.73	0.01	0.59–0.88
deltaMAP	0.54	0.69	0.35–0.72	0.49	0.92	0.32–0.66	0.54	0.61	0.38–0.71
deltaPP	0.55	0.57	0.37–0.73	0.52	0.80	0.35–0.69	0.54	0.64	0.37–0.71

deltaRI, deltaMAP, deltaPP are, respectively, change in resistivity index (*RI*), mean arterial pressure (*MAP*), pulse pressure (*PP*). All these variables are obtained by dividing absolute change in the considered variable by its baseline value. *AUC* area under the curve, *CI* confidence interval

**Table 4:** Diagnostic performances of changes in resistivity index to predict increase in urine output

Increase in urine output	Cut-off on ROC curve	Se	Sp	NPV	PPV	NLR	PLR	Youden’s Indice
0.1 ml/kg/hour	3.3 %	59 %	69 %	62 %	66 %	0.59	1.90	0.28
0.2 ml/kg/hour	3.3 %	63 %	67 %	64 %	67 %	0.55	1.91	0.30
0.3 ml/kg/hour	3.3 %	64 %	65 %	63 %	66 %	0.55	1.83	0.29

Results are expressed as percentages or absolute values as appropriate. *NLR* negative likelihood ratio, *NPV* negative predictive value, *PLR* positive likelihood ratio, *PPV* positive predictive value, *ROC* receiver operating characteristic, *Se* sensitivity, *Sp* specificity

The changes in RI were observed in oliguric and non-oliguric patients (Table 5) with no difference in the magnitude of the effect (interference test; *p* = 0.24). RI decreased significantly in AKI and non-AKI patients (Table 5), with no significant difference in magnitude (interference test *p* = 0.22).

**Table 5:** Changes in hemodynamic variables after fluid challenge according to the presence of oliguria or AKI

	No-oliguria		Oliguria		No-AKI		AKI	
	*N* = 22		*N* = 27		*N* = 11		*N* = 38	
	Before FC	After FC	Before FC	After FC	Before FC	After FC	Before FC	After FC
UO (ml/hour)	53 (37–128)	103 (47–189)	15 (2.5–40)	44 (8–75)	46 (40–102)	86 (49–142)	26 (7–49)	50 (11–104)
RI	0.75 (0.67–0.81)	0.73 (0.65–0.78)	0.74 (0.69–0.80)	0.73 (0.67–0.79)	0.71 (0.61–0.77)	0.68 (0.61–0.74)	0.76 (0.69–0.81)	0.74 (0.66–0.79)
MAP (mmHg)	75 (64–82)	80 (68–94)	69 (64–82)	74 (66–91)	78 (72–94)	90 (78–100)	70 (64–81)	74 (66–90)
PP (mmHg)	50 (44–59)	57 (47–68)	47 (38–63)	52 (47–69)	48 (38–53)	57 (46–63)	48 (42–61)	56 (47–70)

All changes were significant with *p* < 0.01. Of note, interference between subgroups was not significant. Variables are presented as median and interquartile range. *AKI* acute kidney injury, *FC* fluid challenge, *MAP* mean arterial pressure, *N* number of patients, *PP* pulse pressure, *RI* resistivity index, *UO* urine output

A total of 30 (61 %) patients responded to the fluid challenge by an increase of at least 10 % in MAP or PP (Table 6). In these patients, RI decreased from 0.75 (0.67–0.81) to 0.73 (0.66–0.79) (*p* < 0.01). Changes in RI after fluid challenge in patients who were hemodynamic non-responders but had increased urine output are shown in Table 7.

**Table 6:** Changes in resistivity index in responders and non-responders to fluid challenge, defined using a cut-off of a 10 % increase in MAP, PP or both

Fluid responsiveness	Increase in MAP >10 %		Increase in PP >10 %		Increase in MAP or PP >10 %	
	Responsive	Non-responsive	Responsive	Non-responsive	Responsive	Non-responsive
	*n* = 20	*n* = 29	*n* = 27	*n* = 22	*n* = 30	*n* = 19
RI	0.75 (0.70–0.81)	0.75 (0.67–0.80)	0.74 (0.67–0.81)	0.75 (0.68–0.80)	0.75 (0.67–0.81)	0.75 (0.69–0.78)
RI_2_	0.73 (0.67–0.79)	0.73 (0.65–0.78)	0.72 (0.66–0.78)	0.74 (0.66–0.78)	0.73 (0.66–0.79)	0.74 (0.66–0.78)
*p*	<0.01	0.018	<0.01	0.40	<0.01	0.69

Variables are shown as median and interquartile range and were compared using Wilcoxon test. *MAP* mean arterial pressure, *n* number of patients, *PP*, pulse pressure, *RI* resistivity index at baseline, *RI*_*2*_ resistivity index after fluid challenge

**Table 7:** Changes in resistivity index in patients who responded to fluid challenge with increased urine output but were hemodynamic non-responders

Increase in urine output	0.1 ml/kg/hour	0.2 ml/kg/hour	0.3 ml/kg/hour
<10 % increase in MAP			
n/n’ (%)	19/32 (59 %)	16/27 (59 %)	14/25 (56 %)
RI	0.70 (0.67–0.77)	0.69 (0.63–0.74)	0.69 (0.62–0.76)
RI_2_	0.67 (0.63–0.77)	0.67 (0.62–0.73)	0.67 (0.61–0.73)
*p*	<0.01	<0.01	<0.01
<10 % increase in MAP or PP			
n/n’ (%)	12/32 (38 %)	10/27 (37 %)	9/25 (36 %)
RI	0.71 (0.67–0.77)	0.71 (0.65–0.76)	0.72 (0.64–0.77)
RI_2_	0.70 (0.65–0.76)	0.69 (0.64–0.74)	0.67 (0.63–0.75)
*p*	0.182	0.09	0.08

Variables are shown as median and interquartile range and were compared using Wilcoxon test. *MAP* mean arterial pressure, *n*/*n’* number of patients/total number of patients with increased urine output (as a percentage), *PP*, pulse pressure, *RI* resistivity index at baseline, *RI*_*2*_ resistivity index after fluid challenge

## Discussion

This study shows that fluid loading in critically ill patients with acute circulatory failure was associated with significantly decreased RI as assessed by RIAD. In addition, changes in RI were correlated with changes in urine output and better predicted the increase in urine output after fluid administration than changes in MAP and PP at the three urine output thresholds.

These data provide interesting insights into the mechanisms of the renal response to fluids. First, the decrease in RI suggests that renal perfusion increased in these patients. The correlation of changes in urine output with changes in RI but not with changes in MAP or PP suggests that regional factors play a more important role than systemic hemodynamic factors in the regulation of urine production. Direct assessment of intrarenal vasoreactivity may, therefore, better reflect intrarenal pathophysiological features than systemic changes in MAP or PP [18]. Second, renal responders also had a lower RI at baseline, indicating that perfusion pressure and renal perfusion were better preserved in these patients; hence, the effects of fluids may depend on basal renal hemodynamic conditions. Third, an improvement in renal perfusion can be obtained and translated into an increase in urine output even when there are no relevant (<10 %) changes in MAP. This observation emphasizes the relative dissociation of systemic and intrarenal hemodynamics. The limited number of hemodynamic non-responders as assessed by failure to increase MAP or PP may explain the non-significant trend for change in RI observed in this subgroup.

The poor ability of systemic hemodynamic variables to predict an increase in urine output following fluid challenge was notable. Although renal responders had higher baseline MAP, changes in MAP and PP were not associated with renal response to fluids. This observation is consistent with previous investigations by Bourgoin et al. [19] and LeDoux et al. [20], which showed the lack of correlation between changes in MAP and response in urine output [19, 20] or serum creatinine concentrations [19] using norepinephrine to increase MAP from 65 to 85 mmHg. However, Deruddre et al. [8] observed an increase in urine output when MAP was increased from 65 to 75 mmHg using norepinephrine infusion but not when it was increased further from 75 to 85 mmHg. A possible deleterious effect of norepinephrine was considered to explain the observations in the high MAP range. Interestingly, the norepinephrine infusion rate was higher in non-responders than in responders in our renal responsive 0.2 and 0.3 ml/kg/hour groups. Nevertheless, the norepinephrine infusion rate was not correlated with changes in urine output or in RI. The impact of MAP may be affected by individual factors and, although a minimal MAP is required, further increases in MAP are often not associated with improved urine output, creatinine level [21] or renal hemodynamics.

In apparent contrast to our observations, Schnell et al. [9] did not observe significant changes in RI in septic patients who responded to a fluid challenge. However, these authors defined the response to fluid challenge as an increase in descending aortic blood flow evaluated by esophageal Doppler, which is thus a systemic response to fluid challenge, while we preferred to define the response to fluids as an increase in urine output. Hence, their study and ours concur to underline the dissociation between the systemic and the renal response to fluid challenge. Our study further emphasizes that an improvement in renal hemodynamics is essential for the increase in urine output to occur. In addition, we show that changes in MAP and PP, routinely used as surrogates of improved renal hemodynamics, are poorly associated with RI and ineffective at predicting an increase in urine output.

Of note, the changes in renal Doppler variables, although statistically significant, were relatively limited. The observed changes in RI were just above the LSC. Thus, clinical applicability of the observed differences is questionable, especially if multiple and potentially less experienced investigators are performing the examinations [22]. Nevertheless, we showed that, in experienced hands, RI measurements had excellent reproducibility, precision and stability over time. To eliminate any impact of inter-observer variability, one single investigator (MDM) performed all measurements and we suggest that the same sonographer should obtain the before and after measurements. Furthermore, we used strict criteria to define failure in measurement, resulting in more patients being excluded than commonly described. Because of these precautions, the changes observed during fluid challenge were well above the spontaneous variability of measurements.

The technique has some limitations. First, changes in RI may not reflect changes in renal perfusion gradient, as several other confounding factors may be present, including changes in intrarenal compliance, renal interstitial pressure, heart rate, and intra-abdominal pressure. Second, pulsed Doppler techniques provide focal measurements that may not reflect heterogeneous changes in the vascular tone of the entire kidney. In addition, RI measured at the ILA level may not indicate afferent arteriolar or interlobular tone, and these vessels are known to be the most important site of renal myogenic tone regulation [18]. Third, we included patients with no AKI and with different stages of AKI. A recent study on septic patients showed that MAP was correlated with RI in patients with normal renal function but not in those with AKI [23]. In our trial, however, the response to fluids did not differ according to the presence of AKI. We also included septic patients as well as patients with hypovolemic and cardiogenic shock; this heterogeneity may limit the external validity of our results. Fourth, the volume and the type of fluid used may also be confounding factors. The use of colloids in some patients may have affected the oncotic gradient between the two sides of the glomerular filtration barrier and, thereby, reduced the glomerular filtration rate, but the changes in oncotic pressure are likely to have been small. The use of hydroxyethyl starch, even though limited, may also have biased our observations. Finally, we did not measure serum creatinine or urinary chemical content variations, because changes in these variables are slower than urine output, and may be influenced by other factors over time [24, 25].

## Conclusions

We showed that fluid administration reduces intrarenal vasoconstriction and that changes in RI are more effective than changes in MAP and PP to predict an increase in urine output after fluid challenge. Dynamic analysis of intrarenal hemodynamics using RIAD can identify renal responsive patients. However, the use of RI to guide fluid therapy for renal hemodynamic management may be limited by the small magnitude of the changes and potential technical limitations.

## Key messages

- Fluid challenge results in reduced intrarenal vasoconstriction in hemodynamically impaired ICU patients.
- In hemodynamically impaired patients, changes in MAP after a fluid challenge cannot predict an increase in urine output.
- However, changes in renal interlobar artery resistivity index in these patients can predict an increase in urine output.
- Nevertheless, interlobar resistivity index cannot be recommended for routine use, because of the relatively limited magnitude of the changes and need for experienced ultrasonographers.

## Abbreviations

AKI: Acute kidney injury
CE: Coefficient of error
ILA: Interlobar artery
LSC: Least significant change
MAP: Mean arterial pressure
PP: Pulse pressure
RI: Resistivity index
RIAD: Renal interlobar artery Doppler
ScvO_2_: Central venous oxygen saturation
SvO_2_: Mixed venous oxygen saturation
